# Improving the Design of a Conservation Reserve for a Critically Endangered Species

**DOI:** 10.1371/journal.pone.0169629

**Published:** 2017-01-25

**Authors:** Chris Taylor, Natasha Cadenhead, David B. Lindenmayer, Brendan A. Wintle

**Affiliations:** 1 Melbourne Sustainable Society Institute, University of Melbourne, Parkville, Victoria, Australia; 2 School of Biosciences, University of Melbourne, Parkville, Victoria, Australia; 3 Fenner School of Environment and Society, The Australian National University, Canberra, Australian Capital Territory, Australia; Università degli Studi di Napoli Federico II, ITALY

## Abstract

Setting aside protected areas is a key strategy for tackling biodiversity loss. Reserve effectiveness depends on the extent to which protected areas capture both *known* occurrences and areas *likely* to support the species. We assessed the effectiveness of the existing reserve network for Leadbeater’s Possum (*Gymnobelideus leadbeateri*) and other forest-dependent species, and compared the existing reserve system to a set of plausible reserve expansion options based on area targets implied in a recent Population Viability Analysis (PVA). The existing Leadbeater’s Reserve and surrounding reserve system captured 7.6% and 29.6% of cumulative habitat suitability, respectively, across the landscape. Expanded reserve scenarios captured 34% to 62% of cumulative habitat suitability. We found acute trade-offs between conserving Leadbeater’s Possum habitat and conserving habitat of other forest-dependent species. Our analysis provides a template for systematically expanding and evaluating reserve expansion options in terms of trade-offs between priority species’ needs.

## Introduction

A key strategy for tackling biodiversity loss is to set aside protected areas [1,2]. Conservation reserves are an essential part of any comprehensive biodiversity conservation strategy [3,4]. Worboys et al. [1] and Rodrigues and Brooks [5] contend that approximately 25% of the world’s bird biota has been saved from extinction due to conservation reserves. Species distribution models and spatial prioritization algorithms have been key tools in identifying areas in need of protection and in reserve design itself (e.g. [6,7]). Species distribution modelling methods, such as Maxent [8], can be used to identify the extent to which protected areas capture species’ distributions, highlighting gaps in representation [9]. Spatial prioritization software, such as Zonation [10] and Marxan [11], can guide the design of protected areas when complicated trade-offs between species, or between human interests and biodiversity, exist [12]. However, very few studies have explored the adequacy of a reserve system for avoiding extinction of a particular species, despite the expansive literature on conservation reserve design [6,13,14].

Leadbeater’s Possum (*Gymnobelideus leadbeateri*) is listed as critically endangered under Australia’s Environment Protection and Biodiversity Conservation Act (*EPBC Act 1999*). The species has a highly restricted distribution, largely confined to montane ash forests within the Central Highlands region and habitat requirements consisting of old growth and mixed-age forest with hollow-bearing trees [15]. Persistence of remaining populations is threatened by logging and wildfires [16–18]. Special protection zones within forests broadly designated for timber and paper production were established to conserve the species [19]. However, significant habitat loss resulted from the 2009 ‘Black Saturday’ wildfire that burnt approximately 42% of the forest thought to be suitable habitat for Leadbeater’s Possum [20], leaving the species at greater risk of extinction. Repeated field surveys have found the species not occupying areas damaged in that fire, irrespective of the fire severity [17]. Large, old, hollow-bearing trees, on which the species is critically dependent, are also declining rapidly throughout its range [21,22].

A recent Population Viability Analysis (PVA) assessed the long-term viability of populations of Leadbeater’s Possum and suggested that the existing reserve system needed to be significantly expanded [18]. However, the study did not make spatially explicit recommendations about *where* reserve expansion should occur. The effectiveness of the reserve system in conserving Leadbeater’s Possum will depend on the extent to which the reserve area effectively captures both *known* occurrence sites and areas *likely* to support the species, now or in the future.

In this study, we assessed the effectiveness of the existing reserve network for conserving known and likely occurrences of Leadbeater’s Possum and compared the current reserve to a set of plausible reserve expansion options. Because there is significant interest in the degree to which the reserve system caters for other forest-dependent species, we included Greater Glider (*Petauroides volans*), Yellow-bellied Glider (*Petaurus australis*), and Sooty Owl (*Tyto tenebricosa*), which vary markedly in life-history attributes, body size, diet and home range [23]. Although it is relatively abundant throughout its range, Greater Glider is a mostly solitary arboreal marsupial and widely considered to be sensitive to the impacts of forest fragmentation and disturbance [24,25]. The Sooty Owl has a large home range and it occupies hollows in large, old trees [26] that are consequently susceptible to disturbances [27]. The Yellow-bellied Glider has the largest home range of any species of arboreal marsupial in the study area [28]. We analysed how well each species would be represented in reserves arising from single- and multi-species focussed reserve design strategies.

We explicitly analysed the trade-off between obtaining the best possible outcome for a single species, in this case Leadbeater’s Possum, versus achieving an outcome that brings the best balance of habitat provision for multiple species, in this case all four forest-dependent species. The end result was a transparent analysis of where in the landscape reserve expansion should take place for the long-term persistence of Leadbeater’s Possum and how much this will contribute to the conservation of other high-profile forest-dependent species. By integrating the result of a population viability analysis [18] with a spatial prioritization approach, we are able to assess both the adequacy and representativeness of existing and proposed reserve design options; something that is rarely attempted in conservation planning [29].

## Methods

### Study area

Our study region was the Central Highlands of Victoria, located north east of the city of Melbourne in the Australian state of Victoria (37°14' S 144°59' E, 38° 5' S 146°27' E). It includes the Central Highlands Regional Forest Agreement (RFA) area [21], which is approximately 1,100,000 hectares. It comprises 35% is state forest, 16% formal reserves, 4% other public land with the remainder private land [30]. Various kinds of forest covers approximately 64% of the total area of the RFA region [31], including much of mainland Australia’s Mountain Ash forest [32]. These forests provide habitat for a number of threatened and endemic species, but have also been a major source of pulpwood and sawlogs for various industries since the 1930s [33].

Wildfire is the main form of natural disturbance in forests across this area [20]. The impacts of fire are variable, ranging from complete stand replacement after severe fires, to instances where the previous stand survives into the new stand [34,35]. Extensive salvage logging following the 1939, 1983 and the 2009 fires resulted in sizable areas of even-aged stands [36].

**Fig 1:**
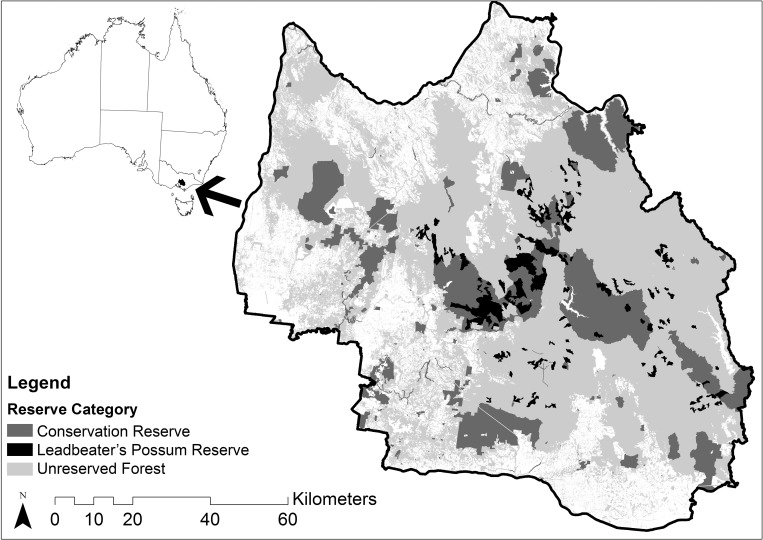
The existing conservation reserve network and the Leadbeater’s Possum Reserve in the study area (Source: [30,37]).

The existing reserve network in the Central Highlands RFA was established over several decades, beginning in 1928 [30]. The current Leadbeater’s Possum Reserve (30,500 ha) was established in 2008, consists of both National Park and State Forest, and is dispersed across the region in small units ranging from 12 ha to 3353 ha [37] (Fig 1).

### Species distribution modelling using MAXENT

Species occurrence data (i.e. presence-only records) for our four target species were sourced from the Victorian Biodiversity Atlas [38]. We used only those records with better than 1km location precision and we removed records at sites where the presence was recorded prior to a fire at that site, since none of our species have been observed at burnt sites post-fire [17]. A suite of biologically-relevant environmental predictors were assembled for each species, which included information on forest type, ecological vegetation class (EVC), disturbance history (logging and fire), regeneration year, forest condition and ranking, where 0 indicated areas that contained no hollow bearing trees, 1 indicated areas with dead hollow bearing trees within regenerating forest, and 2 corresponded to areas with live and dead hollow bearing trees. Bioclimatic data consisted of relevant downscaled ANUCLIM layers [39], as well as layers describing the number of consecutive dry days (<1mm rainfall), elevation relief, 5th percentile of minimum temperature, 95th percentile of maximum temperature and topographic information, such as elevation relief and distance to water bodies [40]. Using the presence-only modelling software Maxent [8] (ver. 3.3.3k), we modelled statistical relationships between environmental predictors and the occurrence records of each species.

Accounting for sampling bias in the spatial distribution of records is particularly important in methods that employ presence-only data [41]. We used a target background sampling approach to account for this kind of bias [41,42], using presence records of Leadbeater’s Possum, Greater Glider, Yellow-bellied Glider, Sooty Owl and public data on other local owl species (see Tables A–E in S1 File).

We constructed models for each of the four target species using the full set of environmental variables. We then modified the models using only those variables which contributed >1% of the explanatory power of the model [40], based on the jack-knifed contributions of each variable. Models were assessed using the area under the receiver operating characteristic curve (AUC) [43] and the AUC_diff_ [44]. AUC is a commonly used measure of predictive performance in presence-only modelling [8] and describes a model’s ability to discriminate between presences and, in this case, background points. For example, if a presence and a background point are drawn at random from the data, the AUC can be interpreted as the probability that the model will have predicted a higher habitat suitability value for the presence location than the background. AUC_diff_ describes the minimum difference between the AUC of the training dataset and that of the test dataset. It gives an indication of whether the model is highly over-fitted. For further information on model settings, see Appendix in S1 File and Table A in S1 File. We converted models into maps of relative likelihood of occurrence for each species across the forested areas in the study region for subsequent use in the spatial prioritization process.

### Spatial prioritization

We used the program Zonation [10,45] (ver. 4.0) to identify priority areas across all forested land within the Central Highlands RFA area. Zonation produces a hierarchical ranking of values over the landscape using a series of algorithms. We used its ‘core area’ algorithm to allocate a conservation value to each cell across the landscape based on: (1) the relative suitability of a cell for each species; (2) the weights assigned to species (see below); and (3) the proportion of the remaining habitat for each species that the cell represents. In this way, Zonation ranked each cell in the landscape according to how ‘irreplaceable’ (*sensu* [46]) it was for achieving representation of suitable habitat for each species. Areas that contained habitat for rarer species tended to be ranked as highly irreplaceable, because habitat for those species is available in few or no other sites in the landscape.

The existing reserve system was used in our analyses in two ways. First, the current Leadbeater’s Possum Reserve was included as the top percentage of ranked cells, allowing us to ascertain where the reserve should be expanded to best complement the existing system and to most efficiently protect Leadbeater’s Possum and the habitat of other forest-dependent species. Second, the existing reserve system was ignored (i.e. the habitat was ranked however the algorithm determined best) allowing us to quantify how efficient the reserve was in protecting Leadbeater’s Possum and other forest-dependent species by comparing it to an ‘optimal’ reserve designed on the basis of their Species Distribution Model (SDM) predictions.

### Species weights

Zonation allows species to be weighted differently, altering the way in which the cells in the landscape are prioritized. The four species were allocated weights in relation to their conservation status according to the IUCN Red List [47] and the Victorian *Flora and Fauna Guarantee Act 1988*. Because there is no best way to weight features, we compared three numerical species weighting scenarios: (1) equal weights (the Zonation default), (2) linear weights, and (3) log weights (Table 1) [48].

**Table 1:** Three weighting schemes for IUCN Red List categories used in this study.

IUCN Red List Category	Species	Equal Weights	Linear Weights	Log Weights
Critically Endangered	Leadbeater’s Possum	1	4	0.5
Endangered	-	1	3	0.05
Vulnerable	Greater Glider, Sooty Owl	1	2	0.005
Near Threatened	Yellow-bellied Glider	1	1	0.0005

Leadbeater’s Possum, Greater Glider and Yellow-bellied Glider are all listed under the IUCN Red List. The Sooty Owl is not currently listed as threatened under the IUCN Red List, but it is listed as ‘threatened’ under the *Flora and Fauna Guarantee Act 1988* [49]. The SDMs for each species were smoothed using a 2D kernel smoothing function based on the species’ home range size. This accounted for the importance of connectivity in a landscape and penalized highly fragmented areas, as all our species have been identified as vulnerable to habitat fragmentation in the study area [50–53].

### Reserve scenarios

We modelled two reserve scenarios based on the recommendations of the PVA by Todd *et al*. (2016). The reserve scenarios were based on the protected area required to achieve a less than 5% chance of the Leadbeater’s Possum population falling to (or below) 500 or fewer adult females in 40 generations. The PVA demonstrated that the smallest assessed threats of a 12.5% habitat decline of hollow-bearing trees and 12.5% habitat burnt in a future fire in 2020 required more than a doubling of the current Leadbeater’s Possum reserve to 67,473 ha (Todd et al., 2016). This provided the basis for reserve scenario 1 modelled in this study. The largest threat modelled by Todd et al. (2016) was a 50% habitat decline of hollow-bearing trees and 50% habitat burnt in a future fire in 2020, which required the Leadbeater’s Possum reserve to be expanded to 171,345 ha. This provided the basis for reserve scenario 2 modelled in this study (Table 2).

**Table 2:** The area of Leadbeater’s Possum Habitat required for a <5% chance of population decline below 500 females under varying scenarios of habitat decline and future fire impact in the Central Highlands over the next 200 years [18]. The ‘habitat decline’ and ‘future fire’ percentage changes indicate possible risks of impact, based on the smallest to largest of threats.

Scenario	PVA Description	Area required (ha)
Existing	Permanent Leadbeater’s Possum Reserve	30,500
Scenario 1	Historical fire + 12.5% habitat decline + 12.5% future fire	67,473
Scenario 2	Historical fire + 50% habitat decline + 50% future fire	171,345

The performance of reserve scenarios was assessed against the existing reserve system by calculating the cumulative habitat suitability value of each species’ distribution layer within the reserve boundaries. The results are expressed as a percentage of the total cumulative value for the region for each target species. The existing Leadbeater’s Possum reserve and formal reserve system are assessed as the baseline. This provides a metric to determine whether the reserves are effective in capturing the areas with the highest habitat suitability values, for example, because reserves containing cells with higher values will not need to be as large as those reserves containing cells of lower values to achieve the same net cumulative value. The scenarios and their respective weights were compared against each other to determine the most efficient trade-offs between the species. For example, will additional protection for the Leadbeater’s Possum result in a larger or smaller negative loss for another target species as different areas are prioritised for their respective scenarios and weights?

## Results

### Species distribution models

**Fig 2:**
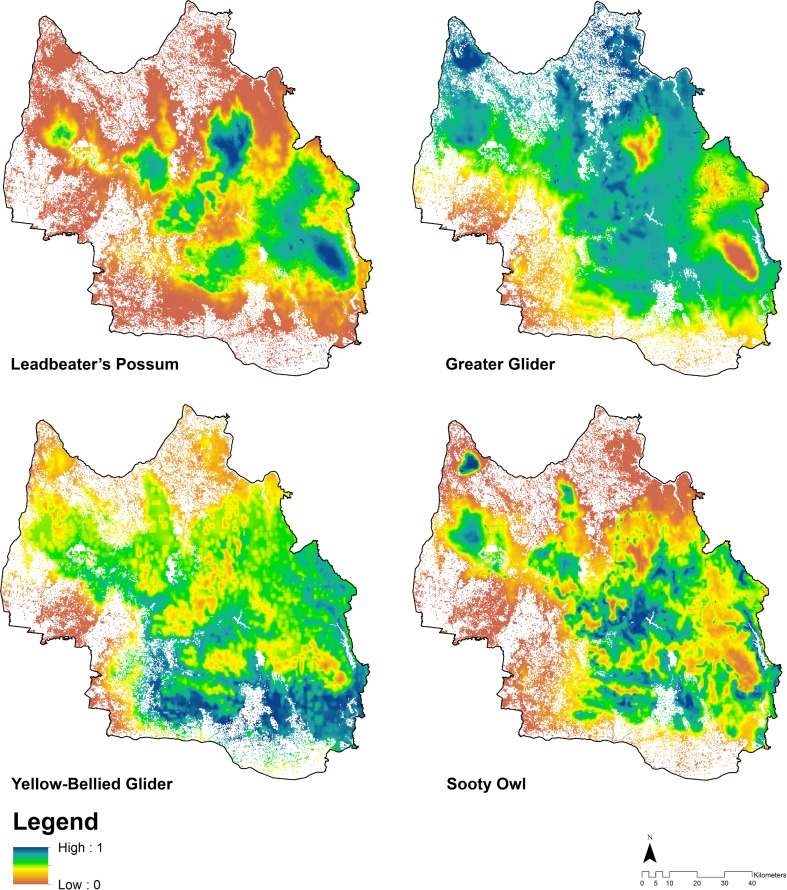
Relative probability of occurrence of Leadbeater’s Possum, Greater Glider, Yellow-Bellied Glider and Sooty Owl in the Central Highlands of Victoria, ranked as values (0–1).

The Maxent models for the four target species performed moderately well in terms of predictive accuracy, with cross-validated AUC values for the Leadbeater’s Possum, Yellow-bellied Glider and Sooty Owl being above 0.7 (see Tables B, D and E in S1 File). However, the Greater Glider performed less well, with an AUC value of 0.63±0.03 (see Table C in the S1 File). The AUC_diff_ values indicate low levels of over-fitting (see Tables B–E in the S1 File). The variables of proportion of old growth forest in a 1-2km radius, temperature seasonality and minimum temperature of the coldest period were considered important variables for all target species. The 5^th^ percentile of minimum temperatures explained the most variance in Yellow-bellied Glider and Sooty Owl models; similarly, the Greater Glider distribution was largely driven by the minimum temperature of the coldest period. Leadbeater’s Possum distribution was driven mostly by the variability of the temperature (temperature seasonality). The relative probability of occurrence of Leadbeater’s Possum, Greater Glider, Yellow-Bellied Glider and Sooty Owl in the study area is shown in Fig 2. Further details of relevant environmental predictors are in Table A in S1 File.

### Performance of the existing reserve system

The existing Leadbeater’s Possum reserve captured only 7.6% of the total cumulative value for the Leadbeater’s Possum. The surrounding formal reserve system captured a larger percentage of the total cumulative value at 29.6% (Table 3). Smaller percentages were found for the other target species. For the Greater Glider, the Leadbeater’s Possum reserve and formal reserve captured 4.2% and 23.8% of the cumulative values, respectively. Similarly for the Yellow-Bellied Glider, the Leadbeater’s Possum reserve and formal reserve captured 3.4% and 24.3%, respectively; and for the Sooty Owl, it was 4.8% and 26%, respectively.

**Table 3:** Percentage of cumulative habitat suitability values captured within the reserve system for Leadbeater’s Possum Reserve and the combined area of the Leadbeater’s Possum Reserve and surrounding reserve system.

Land Tenure	Leadbeater’s Possum (%)	Greater Glider (%)	Yellow-bellied Glider (%)	Sooty Owl (%)
Leadbeater’s Possum Reserve	7.6	4.2	3.4	4.8
Existing Reserve System	29.6	23.8	24.3	26

### Reserve expansion scenarios

**Fig 3:**
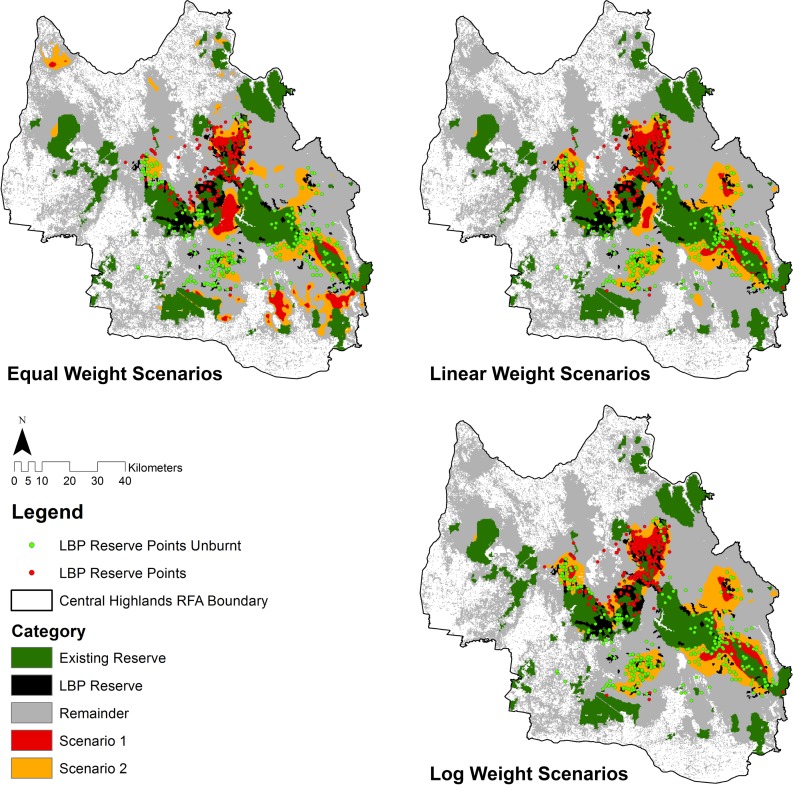
Three Zonation solutions modelling the expansion of the reserve system for Leadbeater’s Possum generated using: (a) equal, (b) linear and (c) log species weighting schemes based on threat status and the Maxent SDMs for each species. The equal weighted scenario resulted in the largest reserve expansion in area under scenario 1 and the linear scenario resulted in the largest area for reserve expansion in area under Scenario 2 (see text).

An expansion of the current Leadbeater’s Possum reserve system was analysed in Zonation using the SDMs for each of the four species under the three different weighting schemes (*viz*: equal, linear, and logarithmic weights). All reserve scenarios prioritised areas outside the existing Leadbeater’s Possum reserve and the remaining formal reserve system (Fig 3). The Equal Weighting identified an additional 23,393 hectares and 95,806 hectares being added to the formal reserve system for Scenarios 1 and 2, respectively. The Linear Weighting identified an additional 22,981 hectares and 102,156 hectares for Scenarios 1 and 2, respectively. The Log Weighting identified an additional 22,450 hectares and 101,400 hectares for Scenarios 1 and 2, respectively.

The reserve scenarios for the Log, Linear and Equal Weighting Zonation solutions increased the cumulative value percentage captured within the reserve system. Under the Log weighting, the Leadbeater’s Possum cumulative value percentage increased from 29.6% under the existing reserve system to 38% and 62% for scenarios 1 and 2, respectively (Table 4). The Linear Weighting was equal for scenario 1 and marginally less at 60% for scenario 2. The Equal weighting was least efficient in capturing modelled distributions for Leadbeater’s Possum at 34% and 49% for scenarios 1 and 2, respectively. The log weighting scenarios were marginally less efficient in capturing the modelled occupancy distribution for the Greater Glider, Yellow-bellied Glider and Sooty Owl, compared with the Linear and Equal Weighting scenarios (Table 5).

**Table 4:** Percentages of the cumulative values for the four target species across all of the cells included in the expanded reserve scenarios.

Weight	Scenario	Leadbeater’s Possum (%)	Greater Glider (%)	Yellow-bellied Glider (%)	Sooty Owl (%)
Equal	Scenario 1	33.7	26.6	28.3	31.0
	Scenario 2	49.2	35.5	38.7	43.5
Linear	Scenario 1	37.7	26.0	27.2	28.9
	Scenario 2	60.0	36.1	37.0	41.4
Logarithmic	Scenario 1	38.1	25.8	27.1	28.4
	Scenario 2	61.5	35.8	36.5	40.0

**Table 5:** Pairwise difference (%) of cumulative values of distributions captured under the reserve scenarios using the different species weighting schemes for each of the target species.

Scenario	Weighting Comparison	Leadbeater’s Possum (%)	Greater Glider (%)	Yellow-Bellied Glider (%)	Sooty Owl (%)
1	Log-Equal	4.4	-0.7	-1.2	-2.5
	Log-Linear	0.4	-0.2	-0.1	-0.4
	Linear-Equal	4.0	-0.6	-1.2	-2.1
2	Log-Equal	12.3	0.3	-2.2	-3.5
	Log-Linear	1.5	9.8	9.2	11.2
	Linear-Equal	10.8	0.6	-1.7	-2.1

The differences for the individual target species between the weighted solutions was greatest for the Leadbeater’s Possum, where there was a 4.4% and 12.3% difference between the Log and Equal weighted solutions for scenarios 1 and 2, respectively. The least difference between weighted solutions for scenario 1 was for the Yellow-bellied Glider, where there was a 0.1% difference between the Log and Linear weighted solutions for Scenario 2, and there was a 0.3% difference between the Log and Linear weighted solutions for the Greater Glider under Scenario 2.

## Discussion

Assessing the design of reserve systems is a key task in conservation science [1,46]. Critical to such assessments is determining how well a protected area conserves a given target taxon and then quantifying how well other species might be conserved by that reserve (e.g. [54]). We have presented a robust method for assessing the effectiveness of the existing reserve system for a critically endangered species (Leadbeater’s Possum) and then conducted a systematic analysis of the trade-offs between conservation priorities for multiple other threatened species. Our approach integrated a systematic analysis of conservation area requirements for ensuring threatened species persistence with spatially explicit recommendations about where to provide extra protection that balances the needs of multiple threatened species; evaluating both conservation adequacy and representativeness together.

We found an acute trade-off between conserving key areas for Leadbeater's Possum and conserving habitat for other forest-dependent species. This arises because their habitat requirements do not strongly overlap. For example, 40% of the Sooty Owl distribution was captured under log weight scenario 2. If equal weights were applied to each species, 44% of the Sooty Owl distribution could be captured under the same scenario. This 4% increase in the representation of the Sooty Owl would come at the cost of a 13% reduction in protection of the distribution of Leadbeater’s Possum. Similar trade-offs are apparent for each of the species pairs under different reserve expansion options and preferences for species weights (Table 5). Given the acute threat to medium-term persistence of Leadbeater’s Possum posed by the combination of fire and logging, it seems appropriate to use the logarithmic weighting scheme presented here or to prioritize solely based on Leadbeater’s habitat, as it affords a higher level of protection to that species. However, a key strength of our approach is that it allows quantification of the implications of such an approach for other species representation in the reserve system. In other ecosystems, that might be characterized by either a greater number of critically endangered species or a more even spread of threat status, it may be more appropriate to employ a flatter weighting such as ‘linear’ or ‘equal weights’ across all species. Our approach to the analysis provides a transparent way to communicate those trade-offs to planners and the public.

Spatial prioritization does not, on its own, provide guidance on how much area should be conserved to ensure persistence of a target species. Population viability analyses can provide these area targets, but PVA alone does not provide spatially explicit recommendations for where additional protected areas should be located. Together, however, as we have shown in this paper, these two approaches can provide informed, species-specific targets for conservation areas and determine where in the landscape they are best placed. Due to the difficulty in obtaining informed conservation area targets, many Zonation studies have relied on arbitrary area thresholds for spatial prioritisation, with only a few studies providing a rationale for setting these thresholds based on analysis of persistence (e.g. [29]). By using PVA to set a biologically-relevant conservation area target for spatial prioritization, there is greater certainty in the effectiveness of targets in ensuring the persistence of species of interest.

We used the best publically-available data to build the SDMs and the recent PVA by Todd *et al*. (2016) to analyze biologically-relevant conservation area targets, improving the levels of certainty in the results. However, there are a number of avenues for furthering this work, should more data become available. Presence-absence data would allow occupancy models to be fitted; which are generally considered to better account for bias and enable better discrimination between suitable and unsuitable habitat [41]. The number of species considered in this study was relatively small, especially compared to many spatial prioritization analyses (e.g. [12]). Other local species could be considered; however, due to the emphasis here on habitat destruction resulting from widespread logging, modelling forest-dependent species known to be impacted by logging [16,18,19] is justified.

PVAs require a large number of difficult-to-obtain parameters to make robust predictions. Todd *et al*. (2016) modelled and provided conservation area targets only for Leadbeater’s Possum. If PVAs were available for all the species in this study, more detailed trade-offs between species could be analyzed and a target-based conservation planning approach, such as Marxan [11], would provide complementary information.

Given the current state of knowledge and the urgency with which decisions about the conservation of a critically endangered species must be made, this study provides crucial and tangible recommendations about where to implement conservation action. The study has also provided insights into the adequacy of the reserve system for protecting a critically endangered species, and set a template for analyzing trade-offs between conservation outcomes for a range of species of interest.

## Supporting Information

S1 File Table A. List of environmental variables used to construct Maxent models of four priority fauna species in the Central Highlands of Victoria. Appendix. Description of the MAXENT models used for the analyses. All models were initially fitted using all available feature types, with 10-fold cross-validation. The background points for the possum and glider species were the presence records of the other two species combined. This is a common approach accounting for bias in presence-only modelling (called Target Group Sampling), where records of species that are surveyed using similar methods can be used as background points. The Sooty Owl background points used presences for all owls in the Central Highlands region that were available on the Atlas of Living Australia (www.ala.org.au), with <1000m accuracy. The models for each species were then refined by removing variables that contributed <1% of the permutation importance in the initial model, and by assessing the most appropriate feature types to capture species’ responses to environmental gradients. Ultimately, all four species’ final models were fitted using only hinge features, which produced complex, smoothed response curves that were easily interpretable. The contribution of each environmental variable included in final MAXENT model for each species is shown in Tables B-E below, alongside the cross-validated test AUC for that model. The mean AUC_diff_ for each model is also shown. AUC_diff_ describes the minimum difference between the AUC of the training dataset and that of the test dataset [8]. This represents another way of assessing the performance of the models; where a smaller AUC_diff_ value indicates a less over-fitted model. Other common validation statistics such as the True Skill Statistic (TSS) [9] were not used as the model predictions were not thresholded (to avoid losing information when it is not necessary [10]) and therefore this statistic is not relevant to this work. All variables listed in Tables B to E contributed >1% permutation importance in the initial model.
(DOCX)
