# Supplementary material for: Improving the Design of a Conservation Reserve for a Critically Endangered Species
Source: PLoS One. 2017 Jan 25;12(1):e0169629. doi: 10.1371/journal.pone.0169629 (PMC5266334; doi:10.1371/journal.pone.0169629)
Supplement: S1 File — Table A. List of environmental variables used to construct Maxent models of four priority fauna species in the Central Highlands of Victoria. Appendix. Description of the MAXENT models used for the analyses. All models were initially fitted using all available feature types, with 10-fold cross-validation. The background points for the possum and glider species were the presence records of the other two species combined. This is a common approach accounting for bias in presence-only modelling (called Target Group Sampling), where records of species that are surveyed using similar methods can be used as background points. The Sooty Owl background points used presences for all owls in the Central Highlands region that were available on the Atlas of Living Australia (www.ala.org.au), with <1000m accuracy. The models for each species were then refined by removing variables that contributed <1% of the permutation importance in the initial model, and by assessing the most appropriate feature types to capture species’ responses to environmental gradients. Ultimately, all four species’ final models were fitted using only hinge features, which produced complex, smoothed response curves that were easily interpretable. The contribution of each environmental variable included in final MAXENT model for each species is shown in Tables B-E below, alongside the cross-validated test AUC for that model. The mean AUCdiff for each model is also shown. AUCdiff describes the minimum difference between the AUC of the training dataset and that of the test dataset [8]. This represents another way of assessing the performance of the models; where a smaller AUCdiff value indicates a less over-fitted model. Other common validation statistics such as the True Skill Statistic (TSS) [9] were not used as the model predictions were not thresholded (to avoid losing information when it is not necessary [10]) and therefore this statistic is not relevant to this work. All variables listed in Tables B to [file pone.0169629.s001.docx]

Supporting Information

Table A. List of environmental variables used to construct Maxent models of four priority fauna species in the Central Highlands of Victoria.

| **Variable name** | **Description** | **Source** |
| --- | --- | --- |
| B04 | Temperature seasonality (standard deviation *100) | ANUCLIM [1] |
| B05 | Max temperature of warmest period (ºC) | ANUCLIM [1] |
| B06 | Min temperature of coldest period (ºC) | ANUCLIM [1] |
| B10 | Mean temperature of warmest quarter (ºC) | ANUCLIM [1] |
| B14 | Precipitation of driest period (mm) | ANUCLIM [1] |
| Dist_to_water | Distance to any permanent water source (decimal degrees) | [2] |
| Dry_runs^†^ | Number of consecutive dry days (with <1mm rainfall) | [3] |
| EVC^*^ | Grouped ecological vegetation classes (1: Wet forest, Montane wet forest, Montane riparian thicket, Sub-alpine treeless vegetation; 2: Sub-alpine woodland, Sub-alpine wet heathland/Alpine valley peatland mosaic; 3: Damp forest; 4: Montane damp forest; 5: Cool temperate rainforest) | Created by CT & NC |
| Prop_forestrank_1km^#^ | Proportion of area containing live & dead hollow-bearing trees within a 1km radius | Created by CT & NC |
| Prop_forestrank_2km^#^ | Proportion of area containing live & dead hollow-bearing trees within a 2km radius | Created by CT & NC |
| Relief | Topographic relief – elevation range (m) | [2] |
| T5^†^ | 5^th^ percentile of minimum temperatures | [3] |
| T95^†^ | 95^th^ percentile of warmest temperatures | [3] |

^*^ Records of Leadbeater’s Possum were recorded in EVCs consisting of Wet Forest, Montane Wet Forest, Montane Riparian Thicket, Sub-alpine Treeless Vegetation, Sub-alpine Woodland, Sub‑alpine Wet Heathland/Alpine Valley Peatland Mosaic, Damp Forest, Montane Damp Forest and Cool Temperate Rainforest.

^#^ Forest condition layers included forest type, ecological vegetation class (EVC), disturbance history (logging and fire), regeneration year, forest condition and ranking (where 0 indicated areas that contained no hollow bearing trees, 1 indicated areas with dead hollow bearing trees within regenerating forest and 2 indicated areas with live and dead hollow bearing trees).

^†^ For the 5th and 95th percentile temperature layers, and the consecutive dry runs layer, daily and monthly climate data were obtained from the Australian Water Availability Project for the period 1977 – 2012 [4,5] at 0.05° spatial resolution (~ 5-km). Temperature data were corrected with an adiabatic lapse rate of 0.00645 °C m-1 [6,7] from the original 0.05° values to a resolution of 0.01° (~1 km) based on a digital elevation model (DEM) resampled from its original 0.0025° to 0.01° resolution (GEODATA 9-second DEM v.3, Geoscience Australia).

Appendix S1: Description of the MAXENT models used for the analyses

All models were initially fitted using all available feature types, with 10-fold cross-validation. The background points for the possum and glider species were the presence records of the other two species combined. This is a common approach accounting for bias in presence-only modelling (called Target Group Sampling), where records of species that are surveyed using similar methods can be used as background points. The Sooty Owl background points used presences for all owls in the Central Highlands region that were available on the Atlas of Living Australia (www.ala.org.au), with <1000m accuracy. The models for each species were then refined by removing variables that contributed <1% of the permutation importance in the initial model, and by assessing the most appropriate feature types to capture species’ responses to environmental gradients. Ultimately, all four species’ final models were fitted using only hinge features, which produced complex, smoothed response curves that were easily interpretable. The contribution of each environmental variable included in final MAXENT model for each species is shown in Tables B to E below, alongside the cross-validated test AUC for that model. The mean AUC_diff_ for each model is also shown. AUC_diff_ describes the minimum difference between the AUC of the training dataset and that of the test dataset [8]. This represents another way of assessing the performance of the models; where a smaller AUC_diff_ value indicates a less over-fitted model. Other common validation statistics such as the True Skill Statistic (TSS) [9] were not used as the model predictions were not thresholded (to avoid losing information when it is not necessary [10]) and therefore this statistic is not relevant to this work. All variables listed in Tables B to E contributed >1% permutation importance in the initial model.

Table B. Leadbeater’s Possum (AUC: 0.77±0.02; AUC_diff_: 0.024±0.027)

| **Variable** | **Permutation importance** |
| --- | --- |
| B10 | 20.8 |
| T95 | 11.4 |
| EVC | 0.9 |
| B04 | 30.2 |
| B06 | 8.1 |
| B14 | 25.2 |
| Relief | 0.9 |
| Prop_forestrank_1km | 2.5 |

Table C. Greater Glider (AUC: 0.63±0.03; AUC_diff_: 0.012±0.035)

| **Variable** | **Permutation importance** |
| --- | --- |
| T5 | 23.8 |
| EVC | 3.3 |
| B06 | 33.5 |
| Dry_runs | 3.2 |
| Prop_forestrank_1km | 1.1 |
| T95 | 11.9 |
| B10 | 22.9 |
| B04 | 0.4 |

Table D. Yellow-bellied Glider (AUC: 0.72±0.04; AUC_diff_: 0.019±0.047)

| **Variable** | **Permutation importance** |
| --- | --- |
| B04 | 11.6 |
| T5 | 28.6 |
| B14 | 17 |
| Dist_water | 4.9 |
| Dry_runs | 12.6 |
| B05 | 15.5 |
| T95 | 3.9 |
| B06 | 4 |
| Prop_forestrank_1km | 1.8 |

Table E. Sooty Owl (AUC: 0.79±0.05; AUC_diff_: 0.012±0.052)

| **Variable** | **Permutation importance** |
| --- | --- |
| T5 | 59.6 |
| Dry_runs | 2.7 |
| B14 | 13.1 |
| Prop_forestrank_2km | 0.4 |
| B04 | 14.9 |
| Relief | 0.9 |
| B06 | 8.5 |

References

1. Xu T, Hutchinson MF (2011) ANUCLIM Version 6.1. Canberra: Fenner School of Environment and Society, The Australian National University. Available: http://fennerschool.anu.edu.au/ research/products/anuclim-vrsn-61.

2. Williams KJ, Belbin L, Austin MP, Stein JL, Ferrier S. Which environmental variables should I use in my biodiversity model? Int J Geogr Inf Syst. 2012;26: 2009-2047.

3. A. Moran-Ordonez (2014) Personal communication.

4. Raupach MR, Briggs PR, Haverd V, King EA, Paget M, Trudinger CM (2009) Australian Water Availability Project (AWAP): CSIRO Marine and Atmospheric Research Component: Final Report for Phase 3. CAWCR Technical Report No. 013. Melbourne: Centre for Australian Weather and Climate Research.

5. Raupach MR, Briggs PR, Haverd V, King EA, Paget M, Trudinger CM. Australian Water Availability Project. 2014. Available: http://www.csiro.au/awap/.

6. Moore JG. The tropospheric temperature lapse rate. Arch Meteor Geophy A. 1956;9(4): 468-470.

7. Sturman A, Tapper N. The Weather and Climate of Australia and New Zealand. Melbourne; New York: Oxford University Press; 1996.

8. Warren DL, Seifert SN (2011) Environmental niche modeling in Maxent: the importance of model complexity and the performance of model selection criteria. Ecol Appl 21:335–342.

9. Allouche O, Tsoar A, Kadmon R (2006) Assessing the accuracy of species distribution models: prevalence, kappa and the true skill statistic (TSS). J Appl Ecol 43:1223–1232.

10. Guillera‐Arroita, G., Lahoz‐Monfort, J.J., Elith, J., Gordon, A., Kujala, H., Lentini, P.E. et al. (2015). Is my species distribution model fit for purpose? Matching data and models to applications. Global Ecology and Biogeography, 24, 276-292.
